# COVID-19 and bilingual children’s home language environment: Digital media, socioeconomic status, and language status

**DOI:** 10.3389/fpsyg.2023.1115108

**Published:** 2023-06-15

**Authors:** He Sun, Justina Tan, Wenli Chen

**Affiliations:** ^1^National Institute of Education, Nanyang Technological University, Singapore, Singapore; ^2^School of Social Sciences, Nanyang Technological University, Singapore, Singapore

**Keywords:** COVID-19, home language environment, child bilingualism, digital media, socioeconomic status, language status, mother tongue language, screen time

## Abstract

Input is considered crucial in bilingual children’s language development. This is especially true for bilingual children’s mother tongue language learning given its common reduction in input opportunities due to the dominance of one language within society, as seen in countries and regions from Wales to Singapore. Previous studies tend to focus on the quantity and quality of conventional active communication and resources (e.g., speaking and reading with parents) on bilingual children’s language development, and substantially, fewer studies have explored this topic from the perspective of digital media. However, the COVID-19 pandemic has accentuated the critical role of digital media in various aspects of life, including bilingual children’s home language environment. Thus, to holistically understand bilingual children’s daily language input patterns, it is imperative to explore both their conventional and digital media input resources. The current study focuses on English-Mandarin bilingual children in Singapore and would like to explore (1) whether their conventional and digital media language environments have been affected by the COVID-19 pandemic and (2) whether the societal status of a language and familial socioeconomic status (SES) would affect bilingual children’s conventional and digital media input. Survey data from 162 parents of English-Mandarin bilingual preschoolers (3 to 6 years old) were used to explore the two research questions. Two online parental questionnaires were employed for data collection. One-way repeated-measures MANOVA and path models were used to address the questions. The results indicated that input patterns from nuclear family members had not been affected by COVID-19; however, the amount and frequency of conventional and digital media materials and activities increased significantly since COVID-19. Higher-SES families possessed more conventional materials and conducted conventional activities more often, while lower-SES families possessed more digital media materials. Both conventional and digital media materials and activities were richer in English than in Mandarin. Higher-SES families perceived digital media usage for learning to be of less importance than lower-SES families. The implications for early bilingual learning following COVID-19 are discussed.

## 1. Introduction

Previous research has established the critical role that the home language environment plays in bilingual children’s language and literacy development (Sun et al., 2018b, 2021, 2022c; Paradis et al., 2020; Sun and Ng, 2021; Song et al., 2022). Most of the existing research tends to examine the impact of the conventional format of language input, such as the current input pattern between parents and children (De Houwer, 2007; Bedore et al., 2016), while much less is known about the use of digital media and its influence on bilingual children’s language learning. In Singapore, a study exploring the relationship between multimedia input and language outcomes of English-Mandarin kindergartners found differential impacts of the resource on English and Mandarin (Sun and Yin, 2020), highlighting the need for greater study in this area. Digital media refers to media content that is produced and provided by digital devices, largely adopting digital media formats of multimedia content (e.g., text, audio, images, and animation) displayed as a single demonstration. The quality of digital media input (e.g., educational value of program content) matters to child development (Courage, 2017) and well-designed educational digital media have been found to positively influence bilingual children’s attention and language learning outcomes (Sun et al., 2019, 2022b). Based on 13 studies that involved 1955 children with a mean age range from 1 to 5 years old, Madigan et al. (2020) found that viewing digital educational programs on television (e.g., Dora the Explorer) has a positive and significant impact on children’s language skills (combined effect size *r* = 0.13). However, screen time and the interactions between parent and child are commonly negatively correlated, especially for low-SES households (Mendelsohn et al., 2008). This has relevance for language development as the use of digital over traditional media has impacted input quantity, with fewer words being exchanged between caregiver and child (Healey et al., 2019). It is thus vital that caregivers remain to be mindful of allowing digital media to replace everyday interactions (Healey et al., 2019), so as to minimize the drawbacks of using digital media. As digital media input (e.g., from TV, tablets, and smartphones) turns to be an increasingly prevalent part of children’s home learning environment since COVID-19 (Sun et al., 2022a), there exists an urgency to adopt a digital media perspective on top of exploring conventional input factors to capture bilingual children’s early language development adequately and holistically. The current study intends (1) to document potential changes in bilingual home language environment since COVID-19 in Singapore, and (2) to explore the relations of familial socioeconomic status (SES) and language status (i.e., societal dominant language vs. mother tongue language) with children’s conventional and digital media input at home.

### 1.1. COVID-19’s impact on home language environment

Since the outbreak of COVID-19, the amount of time that parents spent with their children has increased, giving children more opportunities to receive language input from family members. For instance, in Turkey, the presence of the father at home was particularly notable, leading to a marked increase in language input opportunities for fathers (Kanero and Aktan-Eryciyes, 2021). For bilingual children, COVID-19 may bring differential changes to their dual language environment at home. Sheng et al. (2021) matched two groups of English-Mandarin bilingual families in the US (each cohort *n* = 19, aged 4–8 years old), with one group tested before COVID-19 and one group tested during the pandemic. They found that parents reported speaking less English and more Mandarin at home during the lockdown period. Similarly, a survey conducted on 157 multilingual families from 67 countries found that many of them engaged in more conversation in their minority language at home since the onset of COVID-19, exposing children more frequently to their heritage languages, with some parents even sharing that their children began to grow a liking for these languages that they were previously not so fond of (Murrmann, 2021). Hence, COVID-19 may modify family members’ input pattern in favor of children’s mother tongue language development. Aside from speech patterns, the frequency of literacy activities has also been influenced. Sonnenschein et al. (2021) invited 162 parents of 2- to 9-year-old children in the US to complete a questionnaire on their children’s home literacy and digital environment. They found that as many as “86.3% of the parents reported that their children had increased the use of home literacy activities during COVID-19″ (p. 802). They assumed that the increase was due to the limited opportunities to conduct outdoor activities during the pandemic. Meanwhile, in terms of digital media input, children were seen to engage in more digital activities at home since the pandemic (Murrmann, 2021; Seguin et al., 2021; Sonnenschein et al., 2021). Particularly, there is evidence for an increase in the use of devices to access language-related media (Sheng et al., 2021). Taking shared book reading as an example, research has found that it has shifted toward the use of virtual devices, as caregivers adapt to screen-mediated reading methods, without an overall change to the frequency of shared-book reading practices (Read et al., 2021).

### 1.2. The influence of socioeconomic status and societal language status on language input

Despite the general trend since COVID, there still exists substantial variation in bilingual children’s home language environment. Many factors may contribute to the differences in children’s home language and literacy environments (Sun et al., 2018a,b, 2020), and macro-level issues like socioeconomic status (SES) present a reliable metric to explain some of the differences. SES has commonly been defined using parental levels of education and income (Sun, 2019; Sun et al., 2021, 2022c). Previous research has shown that students from low-income families underperform in language assessments as compared to more affluent peers (Hoff, 2013), pointing toward SES’s impact to either limit or propel children’s language learning. Households with higher SES possess greater access to resources, unlocking the potential for richer home language practice (McDaniel et al., 2017). Apart from being able to provide a greater quantity and variety of books and literacy resources, parents from higher-SES backgrounds may also interact more with their children and tend to use more complex language in their interactions (Hoff, 2006; Ebert et al., 2020). With COVID-19 placing an emphasis on home learning, SES-related differences can potentially widen the divide between students from different SES families. Increased interaction with family at home increases the significance of the impact of quantity and quality of parent–child interaction and the availability of language and literacy resources. This is true for German families, where parents who are less educated were twice less likely to adequately provide support for their children’s schoolwork in terms of engagement and resources during that period (Sari et al., 2021). It is thus worrying to note that parents from lower-SES backgrounds would engage in less formal (e.g., literacy skill practice) and informal (e.g., shared book reading and gameplay) parental practices with their children (Treviño et al., 2021).

Shifting the focus to a bilingual context, English-Spanish children from higher-SES backgrounds also displayed better learning skills in both languages than those from lower-SES families, with this effect being mediated by the home literacy environment (Luo et al., 2021). The SES effect differed for both languages, where the relationship between SES and Spanish knowledge was completely mediated by home literacy environment and Spanish knowledge, whereas for English, there was a direct effect of SES on English learning skills (Luo et al., 2021). In another longitudinal case study, Dolean (2022) illustrated how a higher-SES English-Spanish family was able to extensively support their child’s English learning to the extent where he performed better than his monolingual English peers. To sum up, SES can affect bilingual children’s home language and literacy environment through uneven access to literacy materials at home and the uneven quantity and quality of interaction from caregivers.

Besides SES, bilingual children’s language and literacy environment might be also affected by language status. Bilingual children may have more channels and resources to receive language input in their societal dominant language than in their mother tongue language (Sun et al., 2018a,b, 2020, 2022c; Sun and Yin, 2020). This relates to multilingual countries like Singapore, where there are four official languages (i.e., English, Mandarin Chinese, Malay, and Tamil) and three major ethnic groups (i.e., Chinese, Malay, and Indians). Since 1965, English has been relegated to the societal dominant language for better inter-ethnic communication and trade with the world, being widely adopted in education, business, media, and governance contexts. In contrast, mother tongues (i.e., Mandarin, Malay, and Tamil) are mainly promoted for cultural preservation. The different social statuses of English and mother tongue languages resulted in that “English is increasingly becoming the mother tongue for more and more Singaporeans, and their ethnic languages are technically more like second languages” (Cavallaro and Ng, 2014, p. 36). Under this circumstance, it is unsurprising to witness an increasingly English-dominant environment in more households. Sun and colleagues (2018) examined 805 K1 children’s bilingual home language and literacy environment and found that children’s English input environment was better than their mother tongue language environment in the amount of language input from household members, the amount of children’s language use with household members, the percentage of media input in respective languages, and the number of children’s books. Such discrepancy in children’s dual language environments may be due to parents’ utilitarian thinking and rich resources available in English. As a *lingua franca*, English has been taught and used worldwide, and materials are easy to access in various age-appropriate formats (e.g., books, cartoons, movies, and games). Mother tongue languages, in contrast, may attract substantially less attention from both users and materials developers, resulting in a resource disparity.

### 1.3. The current study

The current study aims to explore the extent to which bilingual children’s home learning environment has been affected by the COVID-19 pandemic, and whether such environment is affected by SES and societal language status. Traditional materials (e.g., hard copy books for children) and activities (e.g., play with magnetic letters or letter toys/cards) were assigned to the conventional group, while those involve digital elements (e.g., “watch educational TV shows or online videos,” “play educational apps on a tablet or smartphone”) were assigned to the digital media category. The possible changes in children’s conventional and digital media input before and since COVID-19 were investigated, at the levels of both English and Mandarin in Singapore. The specific research questions and hypotheses are as follows:

*Research Question 1*: Have English-Mandarin bilingual children’s conventional and digital media language environments been affected by the COVID-19 pandemic in Singapore?

*Hypothesis 1*: Based on the literature review (*e.g*., Sheng et al., 2021; Sonnenschein et al., 2021), both conventional and digital media input environment are expected to be affected. Specifically, children may have an increased proportion of Mandarin input from family members since COVID-19, and they may have more resources and activities at home in both languages.

*Research Question 2*: Do children’s SES and language status influence English-Mandarin bilingual children’s conventional and digital media input environment? SES is operationalized using parental educational level and income.

*Hypothesis 2*: Based on the literature review (*e.g.*, Sun et al., 2018a,b; Luo et al., 2021; Dolean, 2022), children from higher-SES families are expected to have better language environment in terms of resources and activities. Children’s English language and literacy environment is better than that of Mandarin.

## 2. Methods

### 2.1. Participants and procedure

The dataset employed for this study is a part of a longitudinal project on bilingual children’s book reading at home. The project is approved by the university’s institutional review board. Informed consent was obtained on the survey platform before the start of the questionnaires. One hundred and ninety-one parents of preschoolers were recruited by convenience for the COVID-19 questionnaire. Both parent and child had to be living in Singapore since COVID-19 affected the local community to be eligible for the study. The children also had to be English-Mandarin bilingual language learners and have no history of developmental or learning impairment. A total of 26 responses were excluded from the analyses due to the diagnoses of learning/development issues, or due to multiple language exposure (e.g., Filipino dialect, Spanish, and Tamil). Three participants were further excluded due to missing or invalid data (e.g., repeated responses). The final dataset used for analyses consisted of data collected from 162 parents of Mandarin-English bilingual children (86 boys and 76 girls).

### 2.2. Parental questionnaires

Two online parental questionnaires were employed for data collection between April and November 2021 over Qualtrics. This consisted of a questionnaire asking about children’s general background, and a questionnaire specific to COVID-19, which was used in the main analysis. The latter targeted children’s English and Mandarin home language environment along the timeline “before COVID-19” and “since COVID-19.” Items were adapted from the SMALLQ (Chia et al., 2020), the COVID-19-HELP (King et al., 2020), and the QQ-MediaSEED (Sun et al., 2022a). Since the COVID-19 pandemic, people in Singapore mainly experienced two phases of living. In the first phase (April–December 2020), Singaporean first went through a circuit breaker (from April 7, 2020, to June 1, 2020), during which social life and in-person education were suspended, and people remained in a state of uncertainty until the end of that year. Since 2021, the situation improved, but children’s home language and digital environment was still found be heavily influenced by COVID-19, like the first phase (Sun et al., 2022a). Therefore, we combined the two phases and asked parents to indicate the situation during this period which was labeled since COVID-19.

To explore family members’ language input pattern (i.e., father, mother, siblings, maternal grandparents, paternal grandparents, helper, and others), information on the proportion of English and Mandarin spoken by every family member to the child before and since COVID-19 was asked. Given that most of the children in Singapore are from nuclear families (SDS, 2020), the current study focused on the input patterns from the mother, father, and siblings for a measure of core input. The quantity of traditional materials (e.g., hardcopy books for children and educational board or card games) and digital materials (e.g., digital books and digital educational games for children) in English and Mandarin were asked in a similar manner. The frequency of conventional activities and digital media activities was also measured. Examples of the games and programs for digital media were provided. Parents were also invited to indicate their children’s age of first exposure to fixed screens (e.g., TV, desktop computer) and mobile screens (e.g., smartphone, tablet), and the types of digital media they possess at home. Furthermore, parents were asked to indicate how important they felt digital media was for children (i.e., to “Improve language and other skills,” for “Entertainment,” for “Communication” and for themselves (i.e., to “Keep child occupied,” “Distract or divert child’s attention,” “Put child to sleep”). Items related to children and parents were averaged, respectively. Children’s demographic information (e.g., data of birth, gender, mother’s education level) was extracted from another linked questionnaire. To measure SES, information on income and education level was gathered. There were 30 options for monthly household income, ranging from “Below 1,000″ to “15,000 and over,” with S$500 increment for each higher level. There were 8 options for parental education, ranging from “No qualification” to “Doctorate degree.”

### 2.3. Data analysis

One-way repeated-measures multivariate analysis of variance (MANOVA) was adopted to explore the answers for the first research question, and structural equation modeling (SEM; IBM SPSS AMOS 25) was used to examine the postulated relationships in the second research question. SEM is a popular multivariate method commonly used in the social sciences, which leverages on latent trait models. Based on the literature, the models in the current study were created using four measures of fit (Klem, 2000). Chi-square statistics are reported, alongside Tucker and Lewis’s fit index (TLI), comparative fit index (CFI), and the root mean square error of approximation (RMSEA). A non-significant Chi-square suggests that the model in theory is not significantly different from the model derived from data collected, implying good model fit. However, considering that Chi-square is sensitive to sample size, such a result would be challenging to attain. Thus, TLI and CFI values are explored since they are not affected by sample size. A good model fit is suggested by the higher values of these two indicators (≥0.9) (Aryadoust and Liu, 2015). On the other hand, RMSEA values are interpreted by a good model fit being represented by smaller values (≤0.06) (Kenny and McCoach, 2003).

## 3. Results

The descriptive statistics of the 162 children are summarized in Table 1. Children started to receive fixed screen exposure (e.g., TV, computer) since around 1 year old and a half (*M* = 19.83 months, SD = 12.36 months) and they had access to mobile devices (e.g., smartphone and tablet) since around 2 years old (*M* = 26.32 months, SD = 14.27 months). On average, each family possessed three types of digital devices (*M* = 3.4, SD = 0.97), with most indicating ownership of televisions, computers, and mobile devices. Most parents possessed a bachelor’s degree (i.e., 68.10% of mothers and 53.99% of fathers), with a mean household income around Singapore $11000–11,499. In many households, core family members (i.e., father, mother, and siblings) spoke English more often than Mandarin to their children (before COVID-19, *M* = 3.52, SD = 0.94; since COVID-19, *M* = 3.49, SD = 0.94), and the same trend was found in terms of children’s language use with core family members (before COVID-19, *M* = 3.75, SD = 1.01; since COVID-19, *M* = 3.75, SD = 0.96). In terms of conventional materials (i.e., hardcopy books, board/card games), children on average possessed about 10–29 copies of in English (before COVID-19, *M* = 3.09, SD = 0.97; since COVID-19, *M* = 3.33, SD = 1) and 1–9 copies in Mandarin (before COVID-19, *M* = 2.33, SD = 0.81; since COVID-19, *M* = 2.48, SD = 0.82). For children’s digital media materials (i.e., eBooks, Digital educational games), most of the participants either had no such materials at all or had less than 10 copies in English (before COVID-19, *M* = 1.44, SD = 0.66; since COVID-19, *M* = 1.57, SD = 0.68) and in Chinese (before COVID-19, *M* = 1.3, SD = 0.62; since COVID-19, *M* = 1.39, SD = 0.68). In terms of the frequency of conducting traditional activities at home (e.g., playing letter toys), children on average had such activities 1–2 times per week in English (before COVID-19, *M* = 3, SD = 0.94; since COVID-19, *M* = 3.19, SD = 0.97), and 1–3 times per month in Mandarin (before COVID-19, *M* = 2.14, SD = 0.97; since COVID-19, *M* = 2.25, SD = 0.97). For children’s digital media activities at home (e.g., watching educational videos), children on average had such activities 1–3 times per month in English (before COVID-19, *M* = 2.34, SD = 0.97; since COVID-19, *M* = 2.52, SD = 0.98), and barely any digital media activities in Mandarin (before COVID-19, *M* = 1.62, SD = 0.84; since COVID-19, *M* = 1.71, SD = 0.86).

**Table 1:** Descriptive of children’s demographics and bilingual language environment.

		*N*	*M* (SD)		Range	
Age (in months)		162	58.17(6.83)		40–73	
Onset Age. Fixed Screen (in months)		162	19.83(12.36)		0–60	
Onset Age. Mobile Screen (in months)		162	26.32(14.27)		0–60	
Number of Digital Media		162	3.4(0.97)		1–6	
Mother Education Level		162	6.04(0.73)		2–8	
Father Education Level		162	5.85(1.13)		2–8	
Household Income		162	22.01(7.71)		2–30	
	**Before COVID-19**			**Since COVID-19**		
	***N***	***M* (SD)**	**Range**	***N***	***M* (SD)**	**Range**
Language Input. Core Family	162	3.52 (0.94)	1.33–5	160	3.49 (0.94)	1.33–5
Language Output. Core Family	162	3.75 (1.01)	1–5	160	3.75 (0.96)	1–5
Eng. Traditional Materials	162	3.09 (0.97)	1–6	162	3.33 (1)	1–6
Man. Traditional Materials	162	2.33 (0.81)	1–6	162	2.48 (0.82)	1–6
Eng. Digital Media Materials	162	1.44 (0.66)	1–6	162	1.57 (0.68)	1–6
Man. Digital Media Materials	162	1.3 (0.62)	1–6	162	1.39 (0.68)	1–6
Eng. Traditional Activities	162	3 (0.94)	1–5.5	162	3.19 (0.97)	1–5.5
Man. Traditional Activities	162	2.14 (0.97)	1–5.75	162	2.25 (0.97)	1–5.75
Eng. Digital Media Activities	162	2.34 (0.97)	1–6.33	162	2.52 (0.98)	1–6.33
Man. Digital Media Activities	162	1.62 (0.84)	1–5.33	162	1.71 (0.86)	1–5.33
Eng. Digital Media Importance	162	2.46 (0.86)	1–5	162	2.76 (0.89)	1–5
Man. Digital Media Importance	162	2.16 (0.85)	1–5	162	2.34 (0.86)	1–5

### 3.1. Bilingual children’s home language environment before and since COVID-19

One-way repeated-measures multivariate analysis of variance (MANOVA) was conducted to explore the impact of COVID-19 on children’s home language environments. The model for home language environment has nine repeated-measure factors (i.e., Language Input from Core Family, English and Mandarin Traditional Materials, English and Mandarin Traditional Activities, English and Mandarin Digital media Materials, and English and Mandarin Digital media Activities). The results indicated that there was a significant effect of COVID-19 on children’s input environment at home, *F* (9, 144) = 8.487, *p* < 0.0001, Wilk’s Λ = 0.653, partial η^2^ = 0.347. Separate univariate ANOVAs on the outcome variables considering Bonferroni correction (*p* < 0.006) revealed significant improvement on all aspects but not language input pattern from core family members (Table 2).

**Table 2:** The results of “Tests of Within-Subjects Effects” for children’s language environment at home.

Variables	Type III sum of squares	df	Mean square	*F*	*p*	Partial eta squared
Language Input. Core Family	0.01	1	0.01	0.25	0.620	0.002
Eng. Traditional Material	4.47	1	4.47	30.53	0.000	0.167
Man. Traditional Material	1.73	1	1.73	26.22	0.000	0.147
Eng. Traditional Activities	3.09	1	3.09	28.14	0.000	0.156
Man. Traditional Activities	1.00	1	1.00	22.54	0.000	0.129
Eng. Digital Media Material	1.44	1	1.44	33.40	0.000	0.180
Man. Digital Media Material	0.36	1	0.36	12.84	0.000	0.078
Eng. Digital Media Activities	2.69	1	2.69	28.14	0.000	0.156
Man. Digital Media Activities	0.61	1	0.61	15.20	0.000	0.091

### 3.2. SES and language in bilingual children’s home language environment

Table 3 demonstrates the results of the second research question regarding the predictors of home language environment. Children’s parental education levels and household income were used to create the latent “SES” factor. In the SEM model, SES and language (i.e., English vs. Mandarin) were taken as independent variables, while the five environmental factors were taken as dependent variables. The value of each environment factor was the average of the before- and since-COVID-19 pandemic scores. Specifically, SES has been used to predict children’s language input from family members (“Language Input. Core Family. Ave”), quantity of materials (“Traditional Materials. Ave,” “Digital media Materials. Ave”), and types of activities (“Traditional Activities. Ave,” “Digital media Activities. Ave”). Language was used to predict four environmental factors (“Traditional Materials. Ave,” “Digital media Materials. Ave,” “Traditional Activities. Ave,” “Digital media Activities. Ave”). The results reveal that children’s traditional bilingual environment (i.e., language input from core family members, number of traditional materials possessed, and the frequency of conducting conventional activities) are positively related to familial SES: the higher children’s familial SES is, the better their conventional language environment was. In contrast, the amount of digital media materials that a family possessed was significantly and negatively correlated with familial SES. In other words, children from lower-SES families possessed more digital media materials. In terms of language, the results reveal that children possessed more English materials and engaged in more activities in English than in Mandarin, and such “English dominance” applied for both conventional and digital media modalities. The model demonstrated good model fit, *X*^2^(14) = 25.216, *p* = 0.032, CFI = 0.979, TLI = 0.934, RMSEA = 0.05.

**Table 3:** The results of structural equation modeling on children’s bilingual home environment.

Path			*B*	*β*	S.E.	C.R.	*p*
SES	>	Household Income	1.00	0.59			
SES	>	Father Education Level	0.16	0.65	0.02	7.14	^***^
SES	>	Mother Education Level	0.11	0.67	0.02	7.15	^***^
SES	>	Language Input. Core Family. Ave	0.03	0.16	0.01	2.37	0.018
SES	>	Traditional Materials. Ave	0.07	0.31	0.01	4.66	^***^
SES	>	Digital media Materials. Ave	−0.01	−0.14	0.01	−2.07	0.038
SES	>	Traditional Activities. Ave	0.05	0.20	0.01	3.10	0.002
SES	>	Digital media Activities. Ave	−0.01	−0.06	0.01	−0.91	0.362
Language	>	Traditional Materials. Ave	−0.81	−0.42	0.09	−8.69	^***^
Language	>	Digital media Materials. Ave	−0.15	−0.17	0.05	−3.12	0.002
Language	>	Traditional Activities. Ave	−0.90	−0.43	0.10	−8.72	^***^
Language	>	Digital media Activities. Ave	−0.78	−0.47	0.08	−9.57	^***^

^***^p < 0.001, *X*^2^(14) = 25.216, *p* = 0.032, CFI = 0.979, TLI = 0.934, RMSEA = 0.05. B refers to estimate of unstandardized regression coefficients/weights, SE refers to approximate standard error, *β* refers to estimate of standardized regression coefficients/weights, and C.R. refers to critical ratio (*t* value).

We did further analysis with items on parents’ perceived importance of digital media for children (i.e., to ‘Improve language and other skills’, for “Entertainment,” for “Communication” and for parents) (“Keep child occupied,” “Distract or divert child’s attention,” “Put child to sleep”). As with the previous SEM model, the latent “SES” factor and language (i.e., English vs. Mandarin) were used to predict children’s and parental perceived digital media importance. The results (Table 4) reveal that both familial SES and language could significantly affect perceived importance of digital media: the lower the familial SES was, the higher the perceived digital media importance was. In terms of language, both parents and children had higher perceived digital media importance in English than in Mandarin.

**Table 4:** The results of structural equation modeling on perceived digital media importance.

Path			*B*	*β*	S.E.	C.R.	*p*
SES	>	Household Income	1.00	0.59			
SES	>	Father Education Level	0.16	0.66	0.02	7.08	^***^
SES	>	Mother Education Level	0.11	0.66	0.02	7.09	^***^
SES	>	Parent. Importance. Ave	−0.09	−0.39	0.02	−5.33	^***^
SES	>	Child. Importance. Ave	−0.05	−0.25	0.01	−3.57	^***^
Language	>	Parent. Importance. Ave	−0.37	−0.19	0.10	−3.63	^***^
Language	>	Child. Importance. Ave	−0.35	−0.19	0.10	−3.53	^***^

^***^p < 0.001, X2(7) = 4.026, *p* = 0.777, CFI = 1, TLI = 1.027, RMSEA = 0.00.

## 4. Discussion

This study examined (1) whether bilingual children’s home language environment has been affected by COVID-19 and (2) to what extent familial SES and language status (i.e., societal dominant language vs. mother tongue language) would influence children’s home language environment. Based on the literature review and the bilingual situation in Singapore, we proposed the following hypotheses: (1) both conventional and digital media input environment were expected to be affected. Specifically, children might have an increased proportion of Mandarin input since COVID-19, and they might have more digital media resources and activities in both languages and (2) children from higher-SES families were expected to enjoy better language environment in terms of resources and activities, and their English language environment was better than that of Mandarin. Our results have partially confirmed both hypotheses. For the first hypothesis, we found that children had significantly more materials and activities in both languages and both modalities (i.e., for both conventional and digital media) since COVID-19. However, the language input pattern of core family members had not been changed by COVID. For the second hypothesis, we found familial SES indeed impacted children’s dual language environment at home. Higher-SES families held more materials and conducted more activities in the conventional format (e.g., the number of paper books), while lower-SES families possessed more digital media materials. Children in general have significantly more English materials and activities than that of Mandarin Chinese. We discuss our results about the two hypotheses as follows.

### 4.1. The impact of COVID-19 pandemic on bilingual children’s home language environment

Our results for the first research question indicate that COVID-19 has indeed brought changes to bilingual children’s home language environment. Being in line with other studies (e.g., Sheng et al., 2021; Sonnenschein et al., 2021), we also found that there is a significant increase in children’s language input richness. Both in English and Mandarin, children possessed more materials and engaged in more activities, either in the conventional format (e.g., educational board games) or in the digital format (e.g., eBooks). Shift to work-from-home practices allowed parents to spend more time with their children, and many parents can gain a greater awareness of their children’s language status. With this knowledge, they may have turned to educational resources and spent more quality time with their children to boost their children’s language abilities. However, significant increase in digital media resources and activities might also be due to family stress. When parents were fully occupied or felt exhausted, digital devices provided an avenue for children to explore independently, relieving some stress from parental care (Hartshorne et al., 2021). Follow-up studies need to explore parents’ motivation in engaging more digital media devices, distinguishing those who deliberately engage digital resources for educational purposes from those who treat screen devices as convenient babysitters. Parental intentions may lead to different outcomes in children’s language, literacy, and cognitive development. As previous research has pointed out, the effect of digital activity on children’s language skills depends on how engaging the material is. Digital activity that encourages good parent–child interactions as when conventional materials are used are more effective than having a child passively absorb language-relevant information (Florit et al., 2021). As such, promotion of digital activity for children should consider digital interactions that resemble responses and replies. Moreover, the content and the design of digital media also plays a role in its effectiveness in language promotion. For instance, digital books must caution from distracting elements that do not align with the storyline to achieve the same outcomes as printed books (Furenes et al., 2021), reemphasizing the importance of being selective with digital media for improving children’s language skills.

Different from previous findings (i.e., Sheng et al., 2021), however, we have not seen the changes in children’s language input pattern from core family members. In Sheng et al., 2021 study on English-Mandarin bilingual families in the US, Mandarin was found to be used more often between parents and children in the COVID-19 cohort than the pre-COVID cohort, which the authors addressed by identifying congruence with Serratrice’s (2020) finding of “elevated use of the home language in bilingual children of comparable age during lockdown” (p. 7). The discrepancy between our finding and Sheng et al., (2021) might be due to sampling strategy and parental language backgrounds. First of all, Sheng and colleagues compared two groups of children, one before COVID-19 and one since COVID-19, while the current study compared the experiences of one group of children before and since COVID-19. Engaging two samples at different time points bring in additional contextual factors (e.g., employment status) which potentially affect parents’ language use at home. Second, in Sheng et al.’s (2021), study all parents considered Mandarin as the language they spoke when growing up, however, both English and Mandarin could be the dominant language of the young parents in our study, given that bilingual education has been promoted in Singapore since 1967 and many families in Singapore are English-dominant nowadays (Sun et al., 2018b, 2021). Therefore, even though the parents in our study spent more time with their children since COVID-19, they may still prefer to speak in English, and they may persist with this language pattern with their children. In correspondence, their children would respond to their parents in a similar style. As such, Mandarin language use did not see a greater increase as compared to the use of English.

### 4.2. SES and language in bilingual children’s home language environment

Our results for the second research question were aligned with previous findings on the discrepancy between English and Mandarin environment (Sheng et al., 2021; Song et al., 2022). The core family members tended to speak more English than Mandarin to the children, and the households in general possessed significantly more resources in English than in Mandarin. In relation to these findings, children also engaged in more activities in English as opposed to activities in Mandarin. This consistent finding can be attributed to similar reasons other researchers have proposed: the lack of Mandarin learning materials, the social status of English and its importance in the whole education system, and children’s social demand for English with their peers.

Our second hypothesis in relation to familial SES has not been fully supported. Based on the literature, higher-SES families would have more disposable income on various materials, which in turn, would favorably impact their children’s language and literacy development. Therefore, we hypothesized that higher-SES families would have more resources in both conventional and digital media formats. However, this study found that higher-SES families seemed more willing to spend money and time on conventional materials and activities but were reluctant to employ digital media materials. This might be due to parents’ and children’s attitudes toward digital media. The result of parental perceived digital media importance implies that higher-SES families may have less trust or more concerns about using digital media, and they still prefer traditional approaches (e.g., paper book reading) that have stood the test of time, such as magnetic letters games. Moreover, the finding that greater weight was given for the importance of digital media for English use rather than Mandarin use lends support to how English is viewed to hold a stronger status in society. Even though digital media was viewed as more important for English, higher-SES families were still less willing to use these and remained committed to conventional materials.

## 5. Conclusion

There are two critical limitations of the study. First, this is a retrospective study, therefore, parents’ memory of their pre-COVID-19 home language environments might not be accurate. However, given the unpredictable nature of the pandemic, it is not possible to control the nature of the data we have collected. Second, the analysis was based on survey data, and there was no language assessment before the pandemic to allow us to explore the impact of such environment change on children’s dual language performance. However, given the consistent finding on the impact of children’s home language environment on their language and literacy scores worldwide (Paradis, 2011; Paradis et al., 2020) and in the local context (e.g., Sun et al., 2018a,b, 2020, 2022a,b,c; Sun and Yin, 2022), we have good reason to believe that the change of children’s language will cause a ripple effect on their language skills longitudinally. Despite these limitations, the study demonstrates the impact of COVID-19 on children’s language input richness at home. Parents engaged significantly more educational resources and activities since COVID-19, which probably cast a positive impact on children’s language learning. However, the study has also found discrepancies of the richness between languages and families with different SES. It is possible that Singaporean children might receive a boost to their English proficiency and have weaker mother tongue skills in the post COVID-19 era. This deserves more attention from policymakers, educators, and researchers, especially for the preservation of heritage languages.

## Data availability statement

The datasets for this study will not be made publicly available because of the privacy contract signed with the participants. Requests to access these datasets should be directed to the corresponding author.

## Ethics statement

The studies involving human participants were reviewed and approved by Nanyang Technological University. Written informed consent to participate in this study was provided by the participants’ legal guardian/next of kin.

## Author contributions

HS designed the work, analyzed the data, interpreted the results, and wrote the manuscript. JT assisted to analyze the data and contributed to the writing of the introduction section. WC contributed to the writing of the discussion section and helped to proofread the manuscript. All authors contributed to the article and approved the submitted version.

## Funding

This study was funded by Singapore Ministry of Education (MOE) under the Education Research Funding Program (OER 13/19 HS) and administered by National Institute of Education (NIE), Nanyang Technological University, Singapore. Any opinions, findings, and conclusions or recommendations expressed in this material are those of the author(s) and do not necessarily reflect the views of the Singapore MOE and NIE.

## Conflict of interest

The authors declare that the research was conducted in the absence of any commercial or financial relationships that could be construed as a potential conflict of interest.

## Publisher’s note

All claims expressed in this article are solely those of the authors and do not necessarily represent those of their affiliated organizations, or those of the publisher, the editors and the reviewers. Any product that may be evaluated in this article, or claim that may be made by its manufacturer, is not guaranteed or endorsed by the publisher.
